# Identification of a new myotropic decapeptide from the skin secretion of the red-eyed leaf frog, *Agalychnis callidryas*

**DOI:** 10.1371/journal.pone.0243326

**Published:** 2020-12-03

**Authors:** Yitian Gao, Renjie Li, Wenqing Yang, Mei Zhou, Lei Wang, Chengbang Ma, Xinping Xi, Tianbao Chen, Chris Shaw, Di Wu

**Affiliations:** 1 College of Life and Environmental Science, Wenzhou University, Wenzhou, Zhejiang, China; 2 Natural Drug Discovery Group, School of Pharmacy, Queen's University Belfast, Belfast, Northern Ireland, United Kingdom; 3 Chemical Biology Research Center, School of Pharmaceutical Sciences, Wenzhou Medical University, Wenzhou, Zhejiang, China; Fisheries and Oceans Canada, CANADA

## Abstract

Bradykinin-related peptides (BRPs) family is one of the most significant myotropic peptide families derived from frog skin secretions. Here, a novel BRP callitide was isolated and identified from the red-eyed leaf frog, *Agalychnis callidryas*, with atypical primary structure FRPAILVRPK-NH_2_. The mature peptide was cleaved N-terminally at a classic propeptide convertase cleavage site (-KR-) and at the C-terminus an unusual -GKGKGK sequence was removed using the first G residue as an amide donor for the C-terminally-located K residue. Thereafter, the synthetic replicates of callitide were assessed the myotropic activity and showed a significant contraction of balder, with the 0.63 nM EC_50_ value, more potent than most discovered myotropic peptides. The binding mode was further speculated by molecular docking and stimulation. The result indicated that the C-terminal of callitide might selectively bind to bradykinin receptor B2 (BKRB2). Further investigation of the callitide needs to be done in the future to be exploited as potential future drug leads.

## Introduction

The biosynthetic compounds from amphibian skin have attracted considerable attention for many decades and currently, are undergoing a renaissance in interest as chemical drugs fail and new diseases emergence [1]. The bioactive peptides have attracted specific interest due to their potent activities and abundance. The myotropic peptides are significant endogenous peptides in amphibian skin, including bradykinin (BK), bombesin, tachykinin (TK), tryptophyllin (TPH), caerulein, and cholecystokinin (CCK). BKs and BK-related peptides active mammalian BKs, which affect smooth muscle contraction, hypotension, vasodilation, pain, and inflammation. Following the first isolated BK, more than 70 BK-like peptides have further been discovered [2]. Many BK and BK-related peptides, which have identified from anuran species, were isolated from the family *Bombinatatoridae*, *Hylidae*, and *Ranidae* [3–5]. Despite diverse primary sequences, numerous typical BKs are with relatively conservative domains, with the N-terminus/C-terminus of conserved PPGF motif, Arg^1^, and Ser^6^ frequently substitution, the deletion of Arg around N-terminus/C-terminus [6]. Also, some BK variants of anuran often contain the proline residue [7–9].

Earlier research on skin extracts from the arboreal *phyllomedusine* frogs resulted in the identification of many novel peptides such as caerulein, bombesin, sauvagine, tachykinins, and specific opiate receptor ligands. Some of these peptides were later found to be structural analogs of endogenous vertebrate neuro- or regulatory peptides, like cholecystokinin, gastrin-releasing peptide, corticotrophin-releasing factor, and the tachykinins, substance P and neurokinins A and B [10]. While a considerable volume of data has been generated on myotropic peptides in the subfamily *phyllomedusine* frogs, most of these have been reported from species of the genus *Phyllomedusa*, with little information available on the genus *Agalychnis*. Only five tachykinins were reported (AR-1/2/3/4/AL-1) with moderate smooth muscle activities [11]. Here we describe a novel bradykinin-related peptide, callitide, which was identified from the skin secretion of the red-eyed leaf frog, *Agalychnis callidryas*, combining molecular cloning with MS/MS fragmentation. This peptide is different from the classic bradykinin structure, with high sequence similarity to sauvatide [10], a natural peptide identified from *Phyllomedusa sauvagei*. The following smooth muscle pharmacology displayed that callitide specifically stimulated rat urinary bladder smooth muscle with potent activation of contraction in rat urinary balder. The significant target further speculated using molecular docking. Therefore, to study the receptor pathway through which this effect is mediated, provide theoretical support to discover novel endogenous mammalian peptide ligands or for novel drug target elucidation.

## Materials and methods

### Acquisition of skin secretion

Specimens of adult red-eyed tree frogs (n = 3 in each case), *Agalychnis callidryas*, originated in Costa Rica were obtained from a commercial source in the PeruBiotech E.I.R.L., Lima, Santiago de Surco, Peru. They were housed in a purpose-designed terrarium under a 12h/12h light/dark cycle and were fed multivitamin-loaded crickets three times per week. Three months later, skin secretions were obtained by mild transdermal electrical stimulation, washed from the skin with de-ionized water, snap frozen in liquid nitrogen, lyophilized and stored at -20°C prior to analysis, like described before [12,13]. Secretion acquisition was performed under UK Animal (Scientific Procedures) Act 1986, project license PPL 2694 as issued by the Department of Health, Social Services and Public Safety, Northern Ireland. All procedures were vetted by the IACUC of Queen's University Belfast and approved on March 1, 2011.

### Molecular cloning of callitide precursor-encoding cDNA

Five milligrams of lyophilized skin secretion were dissolved in 1 ml of cell lysis/mRNA protection buffer that was obtained from Dynal Biotec, UK. Polyadenylated mRNA was isolated from this by using magnetic oligo-dT Dynabeads as described by the manufacturer (Dynal Biotec, UK). The isolated mRNA was then subjected to 5′ and 3′-rapid amplification of cDNA ends (RACE) procedures to obtain full-length precursor nucleic acid sequence data using a SMART-RACE kit (Clontech UK) as per manufacturer’s instructions. Briefly, the 3′-RACE reactions employed a nested universal (NUP) primer (supplied with the kit) and a degenerate sense primer (S: 5′-TTYGICCIGCIATHYTIGT-3′, R = A + G, Y = C + T, M = A + C, S = C + G), that was complementary to the conservative N-terminal amino acid sequence. The 3′-RACE reactions were purified and cloned using a pGEM-T vector system (Promega Corporation) and sequenced using an ABI 3100 automated sequencer. The sequence data obtained from the 3′-RACE product was used to design a specific antisense primer (AS: 5′-CATGGTGCTCCTCAAATTTATGACA-3′) to a defined site within the 3′ non-translated region of the FK-10-AC-encoding transcript. 5′-RACE was carried out using this primer in conjunction with the NUP primer and resultant products were purified, cloned and sequenced by an ABI 3100 automated sequencer.

### Isolation and identification by reverse-phase high performance liquid chromatography (HPLC) fractionation of skin secretion

Five milligrams of lyophilized skin secretion were dissolved in 0.5 mL of 0.05/99.95 (v/v) trifluoroacetic acid (TFA)/ water and clarified by centrifugation. The supernatant was decanted and directly subjected to reverse phase HPLC fractionation using a Jupiter C-5 analytical column (250 × 4.6 mm). The linear elution gradient employed was formed from 0.05/99.95 (v/v) TFA/water to 0.05/19.95/80.0 (v/v/v) TFA/water/acetonitrile in 80 min at a flow rate of 1 mL/min, followed by isocratic elution (TFA/water/acetonitrile, 0.05/19.95/80.0) for 20 min. The effluent absorbance was monitored by an UV detector at λ = 214 nm, and fractions of 1 mL were collected at minute intervals. Samples (100 μL) of each numbered chromatographic fraction were taken, dried, and stored at -20°C. Furthermore, each fraction was pharmacologically screened the smooth muscle contractile activity. The sample of this fraction that induced a considerable smooth muscle contractile activity was subjected to tandem MS/MS fragmentation sequencing using the LCQ-Fleet electrospray ion-trap MS (Thermo Fisher Scientific). Expected singly-, doubly- and triply- charged b-ion and y-ion fragment m/z ratios were predicted using the MS-Product program available through Protein Prospector (http://prospector2.ucsf.edu/prospector/mshome.htm). The acquired ions were further aligned with predicted ions and the observed fragment ions were indicated.

### Peptide chemical synthesis and purification

The identified mature peptide was acquired using solid-phase peptide synthesis and further purified by HPLC. Peptide samples were synthesized as previously published methods [14,15] using a Tribute Peptide Synthesizer (Protein Technologies, Tucson, AZ, USA). Crude peptides were further purified by reverse-phase HPLC and the purified peptides were confirmed by MALDI-TOF MS.

### Myotropic activity on isolation smooth muscle

Female Wistar rats (250–300 g) were provided by Queen's University Belfast and treated strictly under UK animal research guidelines. All animals were housed at the constant room temperature with a 12/12-h light/dark cycle, fed the standard rodent diet and given water ad libitum. The rats were euthanized by carbon dioxide asphyxiation followed by cervical dislocation then were placed dorsal surface down, and the abdomen was opened utilizing an incision along the midventral line, and subcutaneous fat was carefully dissected. The exposed tissues of bladder, uterus, and ileum were removed and placed in ice-cold Kreb's solution with 95% O_2_, 5% CO_2_. Muscle strips (2 mm × 10 mm) were dissected under a dissection microscope and bathed in Kreb's solution at 37°C with the constant bubbling of 95% O_2_, 5% CO_2_. After a 20 min equilibration period, muscle strips were tested for viability using 60 mM KCl, and the muscle tension was recorded using a transducer (Neurolog 61, Digitimer Ltd., Welwyn Garden, UK). A gradient of peptide concentrations ranging from 10^−11^ to 10^−5^ M in Kreb's solution, were tested on the muscle strips in increasing concentrations at 5 min intervals. Each concentration was applied to six muscle strips. Tension changes were recorded and amplified through pressure transducers that connected to a PowerLab System (AD Instruments Pty Ltd). Data were analyzed to obtain the mean and standard error of responses by Student's t-test, and dose-response curves were constructed.

### Haemolysis assay

The toxicity of peptides was preliminarily investigated using hemolysis assays. For measuring the hemolysis of peptides, peptide dilutions were incubated with 2% horse erythrocytes at 37°C for 2 h. The released hemoglobin was extracted and further detected by the microplate reader at 550 nm.

### Molecular simulation and docking

The molecular simulation and docking were performed for an initial understanding of the binding mode between callitide and its target. I-TASSER webserver was employed to predict and infer the 3-D structural model of callitide [16]. The bradykinin receptor B2 (BKRB2) structure (52–352) was prepared by Joedicke et al. [17] using the software package Rosetta for protein structure prediction [18]. Since BKRB2 belonged to the GPCR family, it was built based on experimentally determined class A GPCR structures as templates and was determined after several rounds of screening [17]. The model quality of callitide and BKRB2 was inspected by z-scores using ProSA [19]. Molecular docking was performed using Rosetta FlexPepDock Server [20,21]. Two hundred high-resolution binding structures were created, and the calculated top prediction with the best affinity score was further rendered with PyMol (Version 1.8 Schrödinger, LLC).

### Statistical analysis

Data were analyzed by the GraphPad Prism software. A two-group comparison was analyzed using a two-tailed unpaired student t-test, and a three-group comparison was analyzed using one-way ANOVA, with post hoc Turkey's multiple comparisons test. A p-value of less than 0.05 was considered significant.

## Results

### Molecular cloning of biosynthetic precursor-encoding cDNA

**Fig 1:**
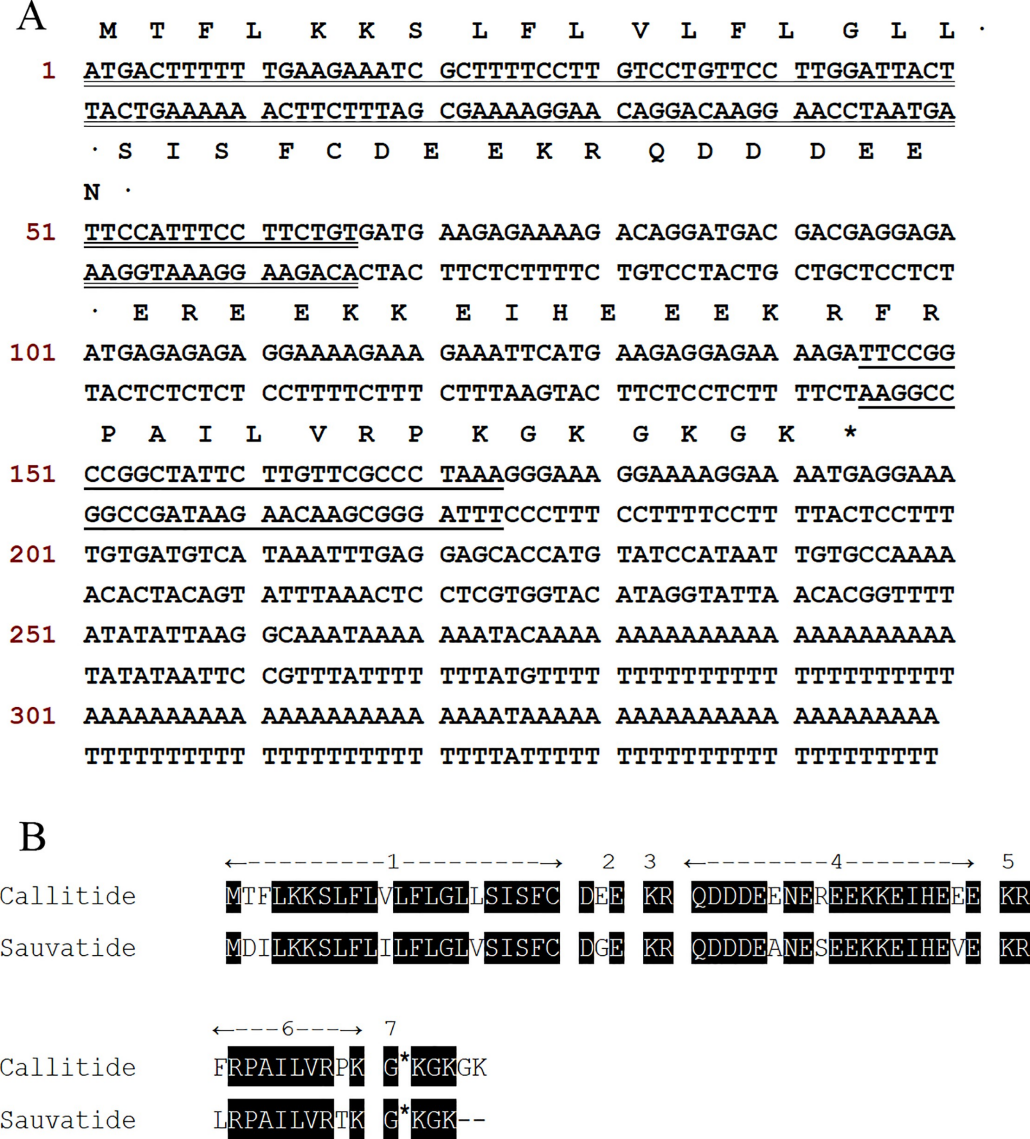
Nucleotide sequence of callitide. **(A)** Nucleotide and translated amino acid sequence of cloned callitide biosynthetic precursor-encoding cDNA. The putative signal peptide is double-underlined, and the mature callitide sequence is single-underlined. The stop codon is indicated with an asterisk; **(B)** Alignment comparison of callitide and sauvatide precursor. (1) Putative signal peptide. (2) Acidic spacer peptide-1. (3/5) Propeptide convertase processing sites. (4) Acidic spacer peptide-2. (6) Mature decapeptide. (7) C-terminal processing site with glycyl (G) residue amide donor indicated with an asterisk.

A single transcript encoding the callitide biosynthetic precursor was consistently cloned from the constructed cDNA library of *Agalychnis callidryas*. The nucleotide sequence and translated amino acid sequence are shown in Fig 1A. The precursor sequence was then aligned using the BLAST from the National Center for Biotechnological Information (NCBI). The alignment results indicated that the open-reading frame consisted of a putative signal peptide (22 residues), a short segment of acidic spacer peptide (3 residues), a classical propeptide convertase processing site (KR), a long segment of acidic spacer peptide (19 residues), a second classical propeptide convertase processing site (KR) and a mature peptide sequence followed by a classical amide-donating G residue for C-terminal amidation (FRPAILVRPK-NH_2_). The sauvatide (CAX48601.1) precursor from *Phyllomedusa sauvagei* displayed the highest (84%) identity, and both shared the same architecture and amidation of the C-terminal residue (Fig 1B). Therefore, the novel peptide was named callitide, and the biosynthetic precursor-encoding cDNA has submitted to the NCBI database (accession number: MT584803).

### Isolation and primary structural validation of callitide from the *Agalychnis callidryas* skin secretions

**Fig 2:**
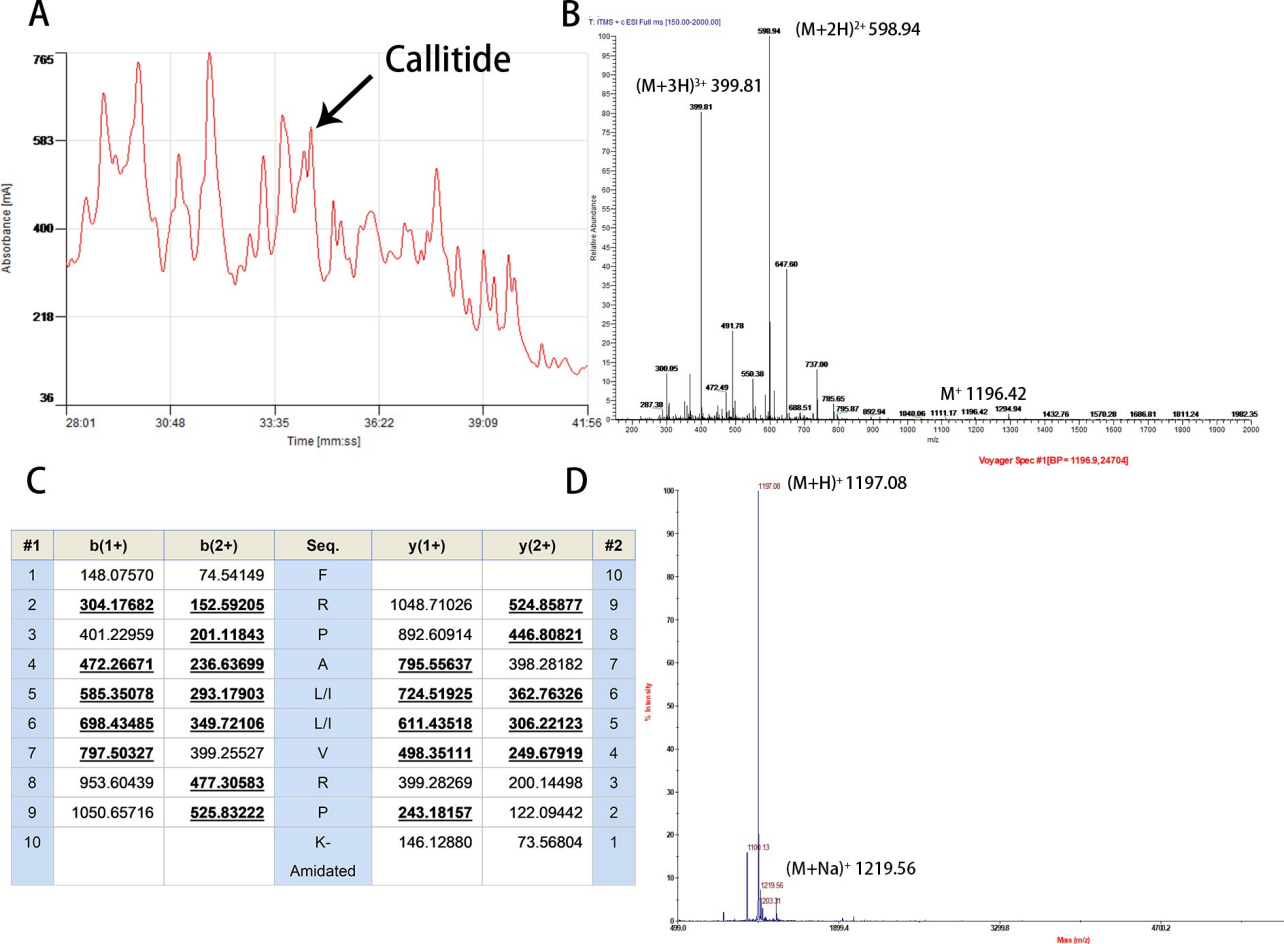
Sepration and identification of callitide mature peptide. **(A)** Region of reverse phase HPLC chromatogram of *Agalychnis callidryas* skin secretion. The arrow indicates the elution position of peak (#35) containing the myotropic decapeptide callitide; **(B)** LCQ mass spectrum of callitide from the skin secretion of *Agalychnis callidryas*. Ions at m/z 598.94 and 399.81 represent doubly charged (M+2H)^2+^ and triply charged (M+3H)^3+^ ions, respectively; **(C)** Electrospray ion-trap MS/MS fragmentation datasets derived from ions corresponding in molecular mass to callitide. Expected singly-, doubly- and triply- charged b-ion and y-ion fragment m/z ratios were predicted and observed fragment ions are indicated in bold type-face and are underlined; **(D)** MALDI-TOF mass spectrum of synthetic copy of callitide.

The crude skin secretion was separated by reverse-phased HPLC in 80 min. Each fraction described aboved was further pharmacologically screened their bladder smooth muscle contractile activity and it revealed that fraction #35 induced a considerable contractilon and the peak was indicated by an arrow in the figure (Fig 2A). Associated with molecular cloning results, a sample of this fraction was subjected to molecular mass analysis using the LCQ system in full scan mode that resolved a primary singly-charged ion at m/z 1196.42. This was subjected to MS/MS fragmentation, ions at m/z 598.94 and 399.81 represent doubly charged (M+2H)^2+^ and triply charged (M+3H)^3+^ ions, respectively (Fig 2B). Furthermore, the singly-, doubly- and triply- charged b-ion and y-ion fragment m/z ratios were predicted by Protein Prospector server and most of the deduced fragment ions were actually observed through the LCQ MS/MS fragmentation (three independent experiments) (Fig 2C). Collectively, the results revealed that the primary structure was unequivocally confirmed from a combination of molecular mass, MS/MS fragmentation data, and molecular cloning data. Once the structure had been established, callitide was synthesized by solid-phase Fmoc methodology, purified by reverse phase HPLC and confirmed by MALDI-TOF MS (Fig 2D).

### Pharmacological effects of callitide

**Fig 3:**
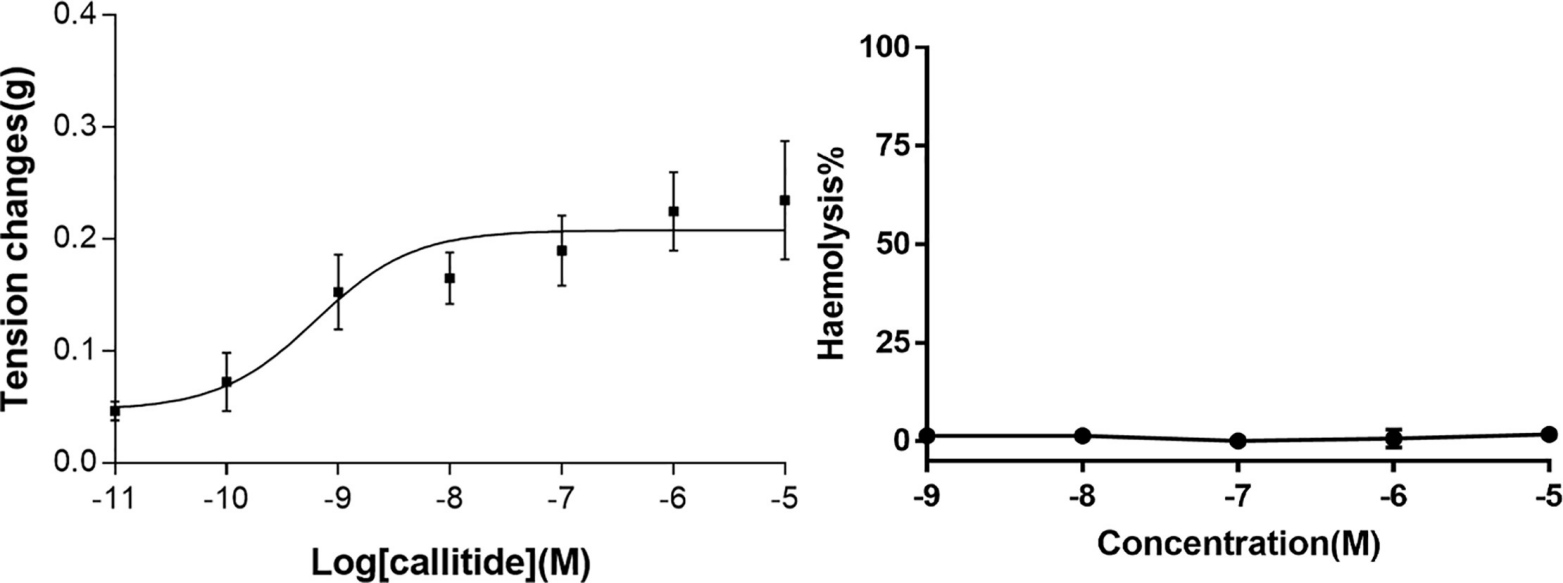
Bioactivities of callitide. **(A)** Dose-response curve of synthetic callitide using rat urinary bladder smooth muscle preparations. Each point represents the mean and standard error of six determinations. EC_50_ = 6.30×10^−10^ M; **(B)** Haemolysis of callitide on horse blood red cells.

Smooth muscle effects of callitide were assessed on different tissues. Callitide displayed significant contractile effects on isolated bladder tissue, while no apparent differences in other tissues were detected. A dose-response curve fitted to the mean of six replicates at each concentration was plotted as tension changes, and the EC_50_ was calculated as 0.63 nM (6.3000×10^−10^ M) (Fig 3A). Besides, hemolysis was preliminarily evaluated the toxicity of callitide. The result indicated that callitide possessed hardly any hemolytic activity at the tested concentration (maximum hemolysis value<3%) (Fig 3B).

### Predicted callitide structure and molecular docking

**Fig 4:**
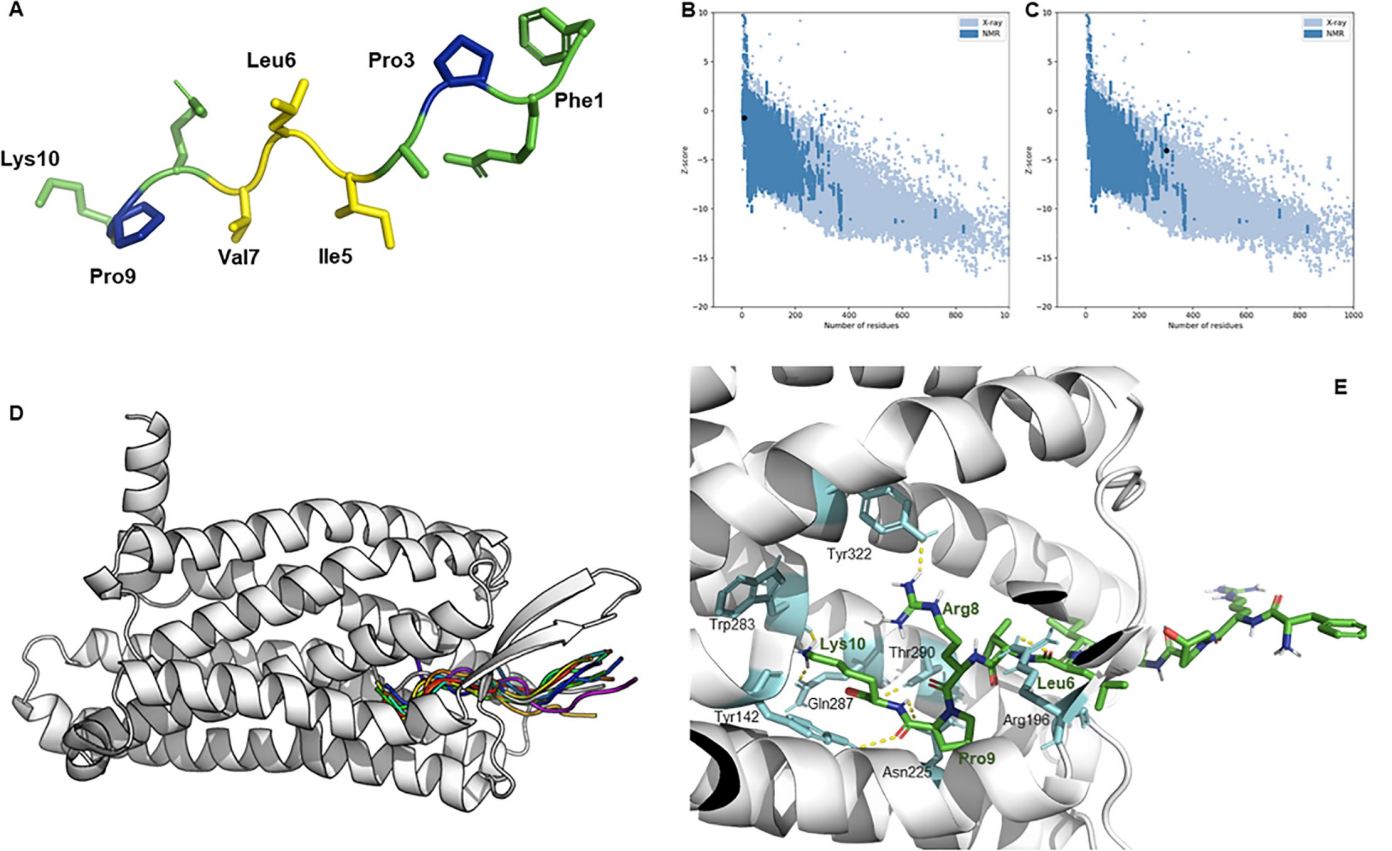
Predicted callitide structure and molecular docking. **(A)** The 3D-model of callitide predicted by I-TASSER. The hydrophobic residues -ILV- in the middle section were colored in yellow while the two proline residues on the flank were colored in blue. **(B, C)** The validation of the predicted callitide (B) and BKRB2 (C) models by z-score using ProSA; **(D)** The peptide callitide cluster in complex with BKRB2. The C-terminus the peptides were inserted into BKRB2, and the peptides were colored; **(E)** The predicted interactions of callitide-BKRB2. Callitide and the receptor residues predicted to be involved in ligand binding were shown as sticks and labeled. Callitide backbone was colored by green with oxygen atoms colored by red and nitrogen atoms colored by blue. The related residues in BKRB2 were colored as cyan, and yellow dot lines indicated the interactions.

The z-score of −0.71(Fig 4B) for the structure of callitide predicted using I-TASSER and the z-score of -4.05(Fig 4C) for the structure of BKRB2 homologically modeled using Rosetta is within the range of scores typically found for protein/peptide native folds identified by NMR with similar size. The model of callitide revealed that the peptide should adopt a strand conformation with a random coil on the flank (Fig 4A). Because of the existence of continuous hydrophobic residues -ILV-, the alkyl side chains pointed to opposite directions one after another. Together with the existing proline residues on both sides, the middle region -PAILVRP- formed a zigzag backbone (Fig 4A). The callitide model was then docked to BKRB2 using Rosetta FlexPepDock. We found that the C-terminal region inserted among the helices of BKRB2 and the C-terminal residues made a significant contribution to the ligand-receptor binding. The carboxyl oxygen and amide hydrogen of Arg10 of callitide formed hydrogen bonds with Thr290 and Asn225 of BKRB2, respectively, and its amine group on its side chain interacted with Trp283 and Gln287. Besides, Pro9, Arg8, and Leu6 showed a connection with Tyr142, Tyr322, and Arg196 of BKRB2, respectively. On the contrary, the N-terminal region of callitide was flexible (Fig 4D and 4E).

## Discussion

The skins of frogs are naked and are directly-exposed to various threats from their environments. Thus all of the compounds in their skin secretions play specific roles in keeping the frogs' safety from the pathogen, predator attack and noxious abiotic factors, or in the regulation of normal biological activities [22]. Studies on leaf frogs from the *Phyllomedusinae* sub-family, have been the subject of intense researchers because of the high level of biochemical diversity in the peptides isolated from their skin secretions over the past decades. The Central American red-eyed leaf frog, *Agalychnis callidryas*, a species of frog belonging to the *Phyllomedusinae*, is characteristic. Unique functional peptides have been discovered and isolated from *Agalychnis callidryas* skin secretions, including opioid peptides (dermorphin, [Hyp^6^] dermorphin), insulin-releasing peptides (CPF-SE3), antimicrobial peptides (DRP-AC1/AC2/AC3, ARP/CRP-AC1, DRP-AC4, medusin AC, plasticin-C1/C2), myotropical peptides (AR-1/2/3/4/AL-1) [1]. Although several types of tachykinin peptides (AR-1/2/3/4/AL-1) have been discovered in this species, few studies have thus far been reported on more novel peptides from its defensive skin secretion. In this study, we have sought the identification of potential novel peptides using a discrete smooth muscle bioassay and were indeed successful in isolating a novel myotropic decapeptide peptide, callitide, and to subsequently determine both its primary and biosynthetic precursor structure using a molecular cloning approach.

Many bradykinins and BRPs have been identified in the skin secretions from amphibians [23–25]. NCBI-BLAST revealed that sauvatide was highly similar to callitide, differing by only two amino acids in the mature peptide sequence. Both of these two myotropic peptide precursors shared the same aspects of post-translational modifications with a glycine acting as an amide donor at the end of the sequences (Table 1). This bioinformatic investigation also provided that a mature peptide sequence contained high similarity with hyposin family identified from *Phyllomedusa hypochondrialis*. Especially for hyposin-4, share high similarity with the callitide, containing an N-terminal tetrapeptide (FRPA), a consistent internal sequence (-VR-) and C-terminal amidation modification. While the C-terminal section of callitide is distinctive. Comparison of the hyposins, the hexapeptide GKGKGK at the C-terminal of callitide was deleted for the amidation throng the post-translational modification. The possibility that exits the post-translational modification of homologous peptide precursors is speculated to species specificity of variation. Extensive trawling of on-line databases failed to provide the function of hyposin family peptides, and preliminary presumed to have antibacterial activity. Besides, the alignment result indicated that the tryptophyllin-1 precursor from *Pachymedusa dacnicolor* and the tryptophyllin-T1 precursor from *Phyllomedusa azurea*, which exhibited similar primary structures to the callitide precursor, except in the mature peptide region [26,27]. While the functions of peptides mainly depend on the mature peptide sequence, therefore the function research failed to give more insights.

**Table 1:** Alignments of mature peptide sequences and origin species.

Peptides	Sequences	Species
*hyposin-1*	LRPAVIRPKGK-NH_2_	*Phyllomedusa hypochondrialis*
*hyposin-2*	LRPAFIRPKGK-NH_2_	*Phyllomedusa hypochondrialis*
*hyposin-3*	LRPAVIVRTKGK-NH_2_	*Phyllomedusa hypochondrialis*
*hyposin-4*	FRPALIVRTKGTRL-NH_2_	*Phyllomedusa hypochondrialis*
*hyposin-5*	LGPALITRKPLKGKP	*Phyllomedusa hypochondrialis*
*HPS-J1*	FRPALIVRTKGK-NH_2_	*Phyllomedusa nordestina*
*sauvatide*	LRPAILVRTK-NH_2_	*Phyllomedusa sauvagei*
*callitide*	FRPAILVRPK-NH_2_	*Agalychnis callidryas*

Hyposin-1 –hyposin-5 [27]; HPS-J1 [28]; Sauvatide [10].

Callitide represents one of the most potent myotropic peptides known for bladder smooth muscle contraction, with EC_50_ values of 0.63 nM. It was more efficient than most other myotropic peptides tested in previous assays within our group, including sauvatide, with an EC_50_ value of 2.2 nM [10]. The myotropic peptide PdT-2 (DMSPPWH-NH_2_), isolated from the skin secretion of the Mexican giant leaf frog, *Pachymedusa dacnicolor*, displayed an EC_50_ value of 4 nM [29]. Kassinin (DVPKSDQFVGLM-NH_2_) and (Thr^2^, Ile^9^)-kassinin (DTPKSDOFIGLM-NH_2_) from the African frog, *Kassina senegalensis*, demonstrated much lower potency in this aspect, with EC_50_ values of 21 nM and 120 nM, respectively [30].

The potent contractile effect of callitide attracted our interest in further investigation of its mode of action. The structure-activity relationship of BRPs has not been studied well, even though a number of BRPs have been reported from amphibian skin [2]. Considering the similar net charge, length, and smooth muscle-contracting activity between callitide and bradykinin, we hypnotize the bradykinin receptor as the target of callitide for a further mode of binding study. Besides, we have reported a new bradykinin family peptide (Ala3, Thr6) bradykinin (RPAGFTPFR) [31] from *Bombina variegate*, and they share a consistent -RPA- short sequence at their N-terminus. For the selection of bradykinin receptors, it was reported that, bradykinin (RPPGFSPFR) is high-affinity ligands for bradykinin receptor B2 (BKRB2) and the removal of the C-terminal arginine made it binds with high affinity to BKRB1 [17]. Furthermore, the C-terminal residue of callitide is lysine, which also shows a positive charge like the arginine residue in bradykinin. Thus, we initially inferred that callitide might selectively bind to BKRB2 if it is indeed a BKR ligand. The callitide-BKRB2 docking result supported our assumption to some extent. The Lys10 residue of callitide contributed the most interactions. For all of the seven polar interactions, Lys10 formed four hydrogen bonds with BKRB2. It might be the critical residue for the ligand-receptor binding. Also, the other three interactions were all contributed by the residues on the C-terminal end (Leu6, Arg8, and Pro9). The N-terminal residues did not form any polar interactions with BKRB2, this region was flexible and away from the receptor, in accordance with the modeling cluster. For the molecular docking study of BK and BKRB2 [17], BK could be constrained into an "S-shape" and all "buried" into BKRB2; thus, the N-terminal residues could also contact with BKRB2. The actions of N-terminal regions differed from ours, further investigation of the callitide real binding mode need to be done in the future.

The callitide thus was identified from *Agalychnis callidryas* for the first time, and it showed potent effects on rat urinary bladder smooth muscle contraction. This result may provide the basis for the discovery of novel endogenous peptide ligands or novel peptide drug development.
